# Integrins Were Involved in Soybean Agglutinin Induced Cell Apoptosis in IPEC-J2

**DOI:** 10.3390/ijms19020587

**Published:** 2018-02-16

**Authors:** Li Pan, Yuan Zhao, Mohammed Hamdy Farouk, Nan Bao, Tao Wang, Guixin Qin

**Affiliations:** 1 Key Laboratory of Animal Production, Product Quality and Security, Ministry of Education, Key Laboratory of Animal Nutrition and Feed Science, Jilin Province, College of Animal Science and Technology, Jilin Agricultural University, Changchun 130118, China; panli0628@126.com (L.P.); zhaoyuan4CL52@126.com (Y.Z.); baonan203@163.com (N.B.); cagewang@163.com (T.W.); 2 Department of Animal Production, Faculty of Agriculture, Al-Azhar University, Nasr City, Cairo 11884, Egypt

**Keywords:** anti-nutritional mechanism, biological functions, cell apoptosis, integrin, soybean agglutinin

## Abstract

Soybean agglutinin (SBA), is a non-fiber carbohydrate related protein and a major anti-nutritional factor. Integrins, transmembrane glycoproteins, are involved in many biological processes. Although recent work suggested that integrins are involved in SBA-induced cell-cycle alterations, no comprehensive study has reported whether integrins are involved in SBA-induced cell apoptosis (SCA) in IPEC-J2. The relationship between SBA and integrins are still unclear. We aimed to elucidate the effects of SBA on IPEC-J2 cell proliferation and cell apoptosis; to study the roles of integrins in IPEC-J2 normal cell apoptosis (NCA) and SCA; and to illustrate the relationship and connection type between SBA and integrins. Thus, IPEC-J2 cells were treated with SBA at the levels of 0, 0.125, 0.25, 0.5, 1.0 or 2.0 mg/mL to determine cell proliferation and cell apoptosis. The cells were divided into control, SBA treated groups, integrin inhibitor groups, and SBA + integrin inhibitor groups to determine the integrin function in SCA. The results showed that SBA significantly (*p* < 0.05) lowered cell proliferation and induced cell apoptosis in IPEC-J2 (*p* < 0.05). Inhibition of any integrin type induced the cell apoptosis (*p* < 0.05) and these integrins were involved in SCA (*p* < 0.05). Even SBA had no physical connection with integrins, an association was detected between SBA and α-actinin-2 ACTN2 (integrin-binding protein). Additionally, SBA reduced the mRNA expression of integrins by down regulating the gene expression level of *ACTN2*. We concluded an evidence for the anti-nutritional mechanism of SBA by ACTN2 with integrins. Further trials are needed to prove whether ACTN2 is the only protein for connecting SBA with integrin.

## 1. Introduction

Soybean agglutinin (SBA), also known as lectin, is a major anti-nutritional factor that represents 5–7% in soybean. As the structure of SBA has stable characteristics [1], such anti-nutritional factor can resist the enzymatic digestion, and induce deleterious toxic or side effects, including influencing immune response (T lymphocytes activation, inflammation and destroying cancerous cells), interaction between cell-to-cell, cell migration, apoptosis, division, cell proliferation, and signal transduction [1,2,3].

SBA is a non-fiber carbohydrate related protein and has a specific binding with the gastrointestinal tract [4], this mainly depends on the glycosylation (including glycoproteins, glycolipid and high glycosylation mucoprotein) in the intestinal epithelium [5]. The specific binding of SBA to the intestinal epithelial cells surfaces is the precondition for deleterious toxic or side effects [6]. For example, weaned pigs supplemented high levels of SBA can bind to intestinal epithelial cell, then reduce the epithelial tight junction protein (occludin) expression and increase the mucosal permeability [7].

Integrins are a family member of type-I transmembrane heterodimeric glycoprotein receptors for cell adhesion [8]. Such transmembrane receptors regulate cell-to-cell and cell-to-extracellular matrix interactions and signals [9,10,11]. At the cellular level, functions of integrins are related to mechanical links in cell adhesion, cell migration, cell signal transduction, cell proliferation and cell apoptosis [12,13,14]. Integrins are glycoproteins which are important for ligand binding. The intracellular cytoplasmic domains of integrins can associate directly with many cytoskeletal proteins and intracellular signal molecules [12,14].

The related researches indicated that SBA can inhibit cell proliferation and induce cell apoptosis [1,15], however the mechanism has not been reported. Our former research has identified five integrin subunits (α2, α3, α6, β1 and β4) that specially expressed in porcine intestinal columnar epithelial cells (IPEC-J2), and integrins α2, α6, and β1 were involved in SBA-induced cell cycle alteration in IPEC-J2 [15]. However, no study has reported whether integrins are also involved in SBA-induced cell apoptosis (SCA) in IPEC-J2. Additionally, the relationship and connection type between SBA and integrins are still unclear.

Therefore, this research aims to determine the effects of SBA on IPEC-J2 cell proliferation and cell apoptosis; to study the roles of integrins in the process of SCA; and to illustrate the interaction and connection type between SBA and integrin subunits in IPEC-J2. The current research provided a basic for the research of the SBA anti-nutritional mechanism and we suggested that integrins can be used as a new research idea to improve SBA-induced cell biological functions alterations. The current study provided an important evidence and theoretical basic for revealing the anti-nutritional mechanism of SBA by α-actinin-2 (ACTN2) with integrins.

## 2. Results

### 2.1. SBA Decreased IPEC-J2 Cell Proliferation Detected by 3-(4,5-Dimethylthiazol-2-yl)-2,5-Diphenyltetrazolium Bromide (MTT) Assay

**Figure 1:**
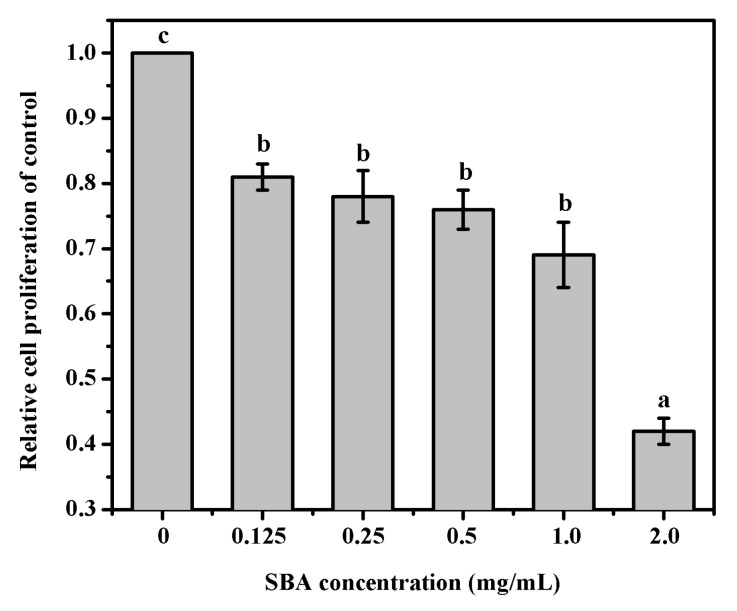
Effects of SBA on IPEC-J2 proliferation. Cell proliferation was measured by MTT assay at 6 concentrations points (0, 0.125, 0.25, 0.5, 1.0, or 2.0 mg/mL) for 24 h. Absorbance was measured at 570 nm. Means with different superscript are significantly different in compare with its control. Data are represented as mean ± standard error of mean (SEM) (*n* = 3).

MTT was performed to determine cell proliferation in IPEC-J2. The results indicated that after being treated with 0, 0.125, 0.25, 0.5, 1.0, or 2.0 mg/mL SBA for 24 h, IPEC-J2 cell proliferation was significantly lower (*p* < 0.05, Figure 1). When the cells were treated with 2.0 mg/mL SBA, cell proliferation (%) was lower to the lowest level, when compared with other SBA treatments (*p* < 0.05).

### 2.2. Morphometric Analysis by Contrast Microscopy

**Figure 2:**
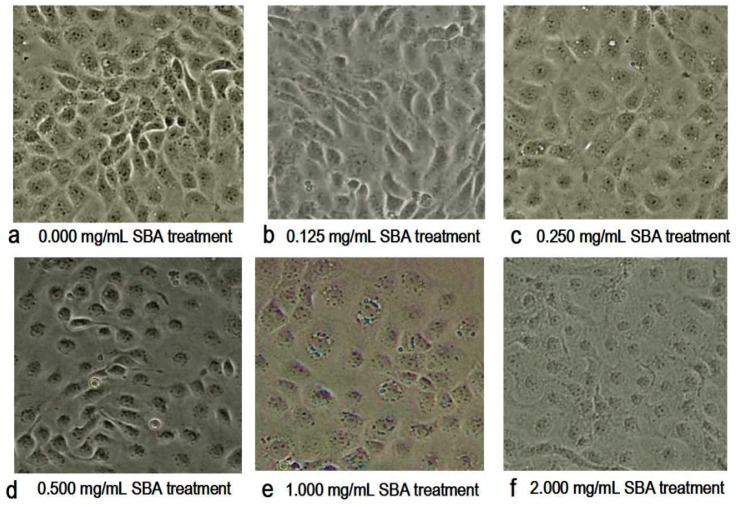
(**a**–**f**) Effect of soybean agglutinin (SBA) on the morphology of IPEC-J2 cells (200×). IPEC-J2 was cultured with 0, 0.125, 0.25, 0.5, 1.0 or 2.0 mg/mL SBA for 24 h. Cell morphology was observed in different treatments by contrast microscopy at 200× magnifications. (**a**) Control, 0.000 mg/mL SBA treatment; (**b**) 0.125 mg/mL SBA treatment; (**c**) 0.250 mg/mL SBA treatment; (**d**) 0.500 mg/mL SBA treatment; (**e**) 1.000 mg/mL SBA treatment; (**f**) 2.000 mg/mL SBA treatment.

With the increased concentration of SBA, the morphology and the density of the cells were changed obviously as shown in Figure 2. The main morphological differences in SBA treatments were the decreased cell numbers and the ambiguous boundaries between adjacent cells, when compared with control. Therein, 2.0 mg/mL SBA had the most significant effects on the morphology of IPEC-J2.

### 2.3. SBA Induced IPEC-J2 Cell Apoptosis

The effects of SBA on IPEC-J2 cell apoptosis were analyzed by the determination of the fraction of cells positive for active caspase-3 in different SBA treatments using flow cytometry (FCM) and the determination of Bcl-2 relative mRNA expression using quantitative real-time polymerase chain reaction (qRT-PCR).

**Figure 3:**
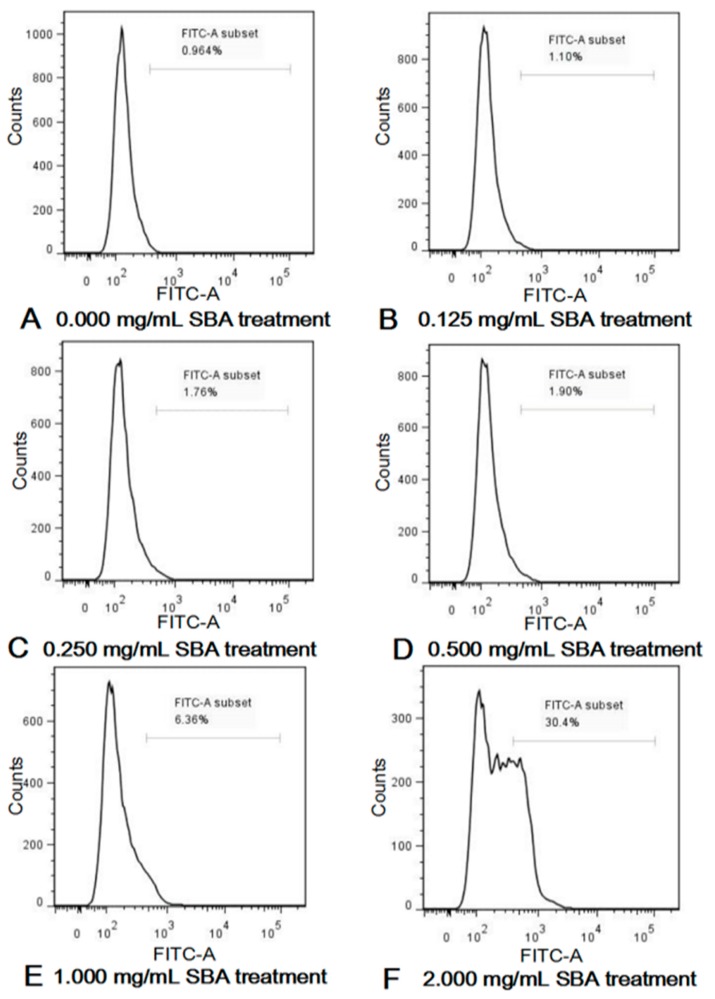
(**A–F**) SBA induced cell apoptosis in IPEC-J2. IPEC-J2 was cultured with 0, 0.125, 0.25, 0.5, 1.0 or 2.0 mg/mL SBA for 24 h. Cell apoptosis was determined by FCM and shown in Control, 0.000 mg/mL SBA treatment (**A**); 0.125 mg/mL SBA treatment (**B**); 0.250 mg/mL SBA treatment (**C**); 0.500 mg/mL SBA treatment (**D**); 1.000 mg/mL SBA treatment (**E**); 2.000 mg/mL SBA treatment (**F**).

Active caspase-3 is a marker for the cells undergoing apoptosis. After incubation with different concentrations of SBA (0, 0.125, 0.25, 0.5, 1.0, and 2.0 mg/mL) for 24 h, effects of SBA on IPEC-J2 apoptosis were determined using FCM. As shown in Figure 3 and Figure S1, SBA with lower concentrations (0.125, 0.25 and 0.5 mg/mL SBA) did not induce cell apoptosis (*p* < 0.05). When the concentrations reached to a certain level (1.0 and 2.0 mg/mL SBA), fraction of cells that positive for active caspase-3 in these two SBA treatment groups were significantly higher than the control (*p* < 0.05). When the cells were treated with 2.0 mg/mL SBA, cell apoptosis (%) was increased to the highest level, compared with other SBA treatments (*p* < 0.05). Therefore, 2.0 mg/mL of SBA was selected as the inflection point in the next experiment, as this concentration provided the highest cell apoptosis rate than the other SBA levels.

**Figure 4:**
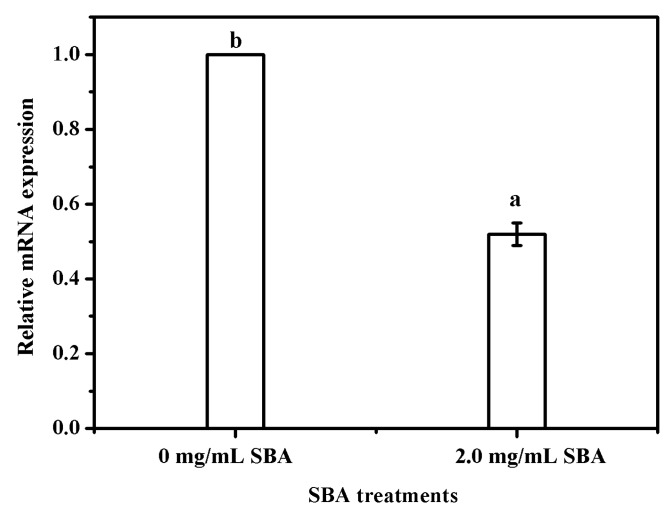
SBA lowered the mRNA expression of Bcl-2 in IPEC-J2. The cells were incubated with 0 or 2.0 mg/mL SBA for 24 h, the effects of SBA on mRNA expression of Bcl-2 were analyzed using qRT-PCR. Means with different superscript are significantly different in compare with its control. Each column is depicted as a mean ± SEM of three independent experiments.

Bcl-2 (B-cell lymphoma 2) is a member of the Bcl-2 family of regulator proteins that regulate cell death (apoptosis), and is specifically considered as an important anti-apoptotic protein. Subsequently, the effects of SBA on Bcl-2 mRNA expression was determined using qRT-PCR and the results indicated that 2.0 mg/mL SBA significantly (*p* < 0.05) lowered the mRNA expression of Bcl-2 (Figure 4).

### 2.4. The Integrins Were Involved in SBA-Induced IPEC-J2 Cell Apoptosis

**Figure 5:**
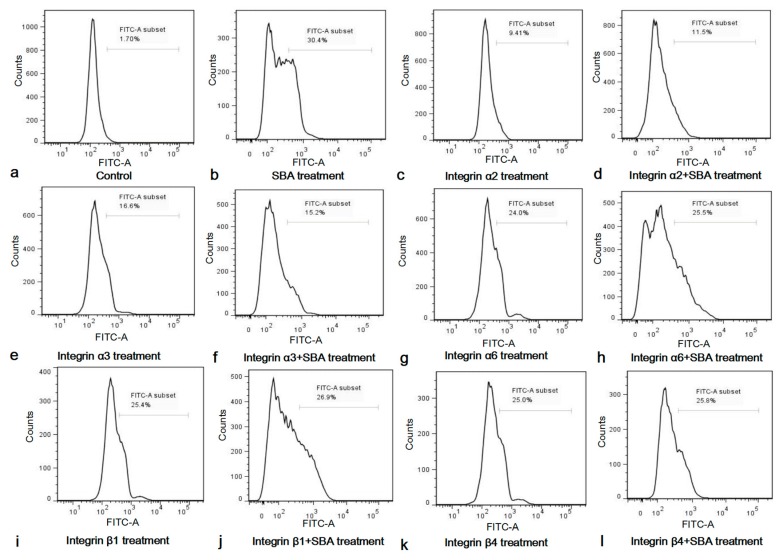
(**a**–**l**) Functions of integrin inhibitors on IPEC-J2 cell apoptosis and SCA. Cell apoptosis in different treatments for 24 h was determined by FCM and shown in (**a**) Control, 0.0 mg/mL SBA treatment; (**b**) 2.0 mg/mL SBA treatment; (**c**) α2 inhibitor treatment; (**d**) α2 inhibitor + 2.0 mg/mL SBA treatment; (**e**) α3 inhibitor treatment; (**f**) α3 inhibitor + 2.0 mg/mL SBA treatment; (**g**) α6 inhibitor treatment; (**h**) α6 inhibitor + 2.0 mg/mL SBA treatment; (**i**) β1 inhibitor treatment; (**j**) β1 inhibitor treatment + 2.0 mg/mL SBA treatment; (**k**) β4 inhibitor treatment; (**l**) β4 inhibitor treatment + 2.0 mg/mL SBA treatment.

The 10 μg/mL of integrin inhibitors [15], 2.0 mg/mL SBA or both concentrations were selected to determine the roles of integrin inhibitors on cell apoptosis and SCA in IPEC-J2 by FCM (Figure 5).

After stimulation with integrin inhibitors for 24 h, the apoptosis rate was significantly increased, when compared with control (*p* < 0.05, Figure S2). This indicated that integrins α2, α3, α6, β1 and β4 were important for IPEC-J2 cell apoptosis, as the functional inhibition of any integrins can lead to cell apoptosis in IPEC-J2. The apoptosis rates in integrin inhibitor treatment groups were lower than 2.0 mg/mL SBA treatment group (*p* < 0.05).

Since SBA can induce IPEC-J2 cell apoptosis, and integrins play an indispensable role in the apoptosis of IPEC-J2, we speculated that integrins may be involved in the process of SCA. To further demonstrate the important roles of integrin in this process, the percentage of the apoptosic cells in integrin inhibitor groups and integrin inhibitor + 2.0 mg/mL SBA treatment groups were compared and analyzed (Figure S3). The results showed that apoptosic cells in integrin inhibitor α2, α3, α6, β1 or β4 treatment had no significant differences when compared with their relevant integrin inhibitor + 2.0 mg/mL SBA treatment (*p* > 0.05), but such apoptosic cells in integrin inhibitor treatments were lower than 2.0 mg/mL SBA treatment (*p* < 0.05). These results suggested that SBA no longer caused apoptosis in addition of integrin α2, α3, α6, β1 or β4 inhibitor. Therefore, integrins were involved in SCA in IPEC-J2.

### 2.5. SBA Lowered the mRNA Expression of Integrins Detected by qRT-PCR

**Figure 6:**
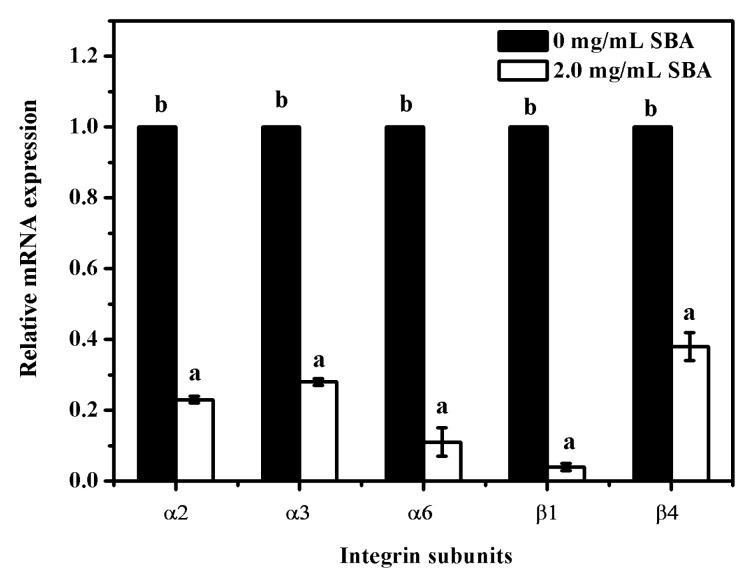
Relative mRNA expression of integrin subunits after stimulation by SBA. The mRNA levels of integrins (α2, α3, α6, β1, β4) were measured by qRT-PCR when IPEC-J2s were treated with 0 or 2.0 mg/mL SBA for 24 h. Means with different superscripts are significantly different in compare with its control. Each column is depicted as a mean ± SEM of three independent experiments.

To determine the relationship between SBA and integrins (α2, α3, α6, β1 and β4) mRNA expression, mRNA levels of integrins were determined after stimulation with 0 or 2.0 mg/mL SBA for 24 h using qRT-PCR. The results showed that the mRNA expression reduction was observed in α2, α3, α6, β1 and β4 (*p* < 0.05), when compared with control (0 mg/mL SBA treatment, Figure 6). Therefore, SBA had a negative effect on integrin α2, α3, α6, β1 and β4 mRNA levels.

### 2.6. Identification of the Connection Type between SBA and Integrins in IPEC-J2

For further estimating the connection type between SBA and integrins (direct connection or indirect connection), all the SBA-binding proteins on IPEC-J2 cell membranes were separated using co-immunoprecipitation and identified with mass spectrometry (Q–E). Direct connections between SBA and integrins have been indicated, when integrin subunits were appeared in the result of SBA-binding proteins. Otherwise, they had no direct association between them.

**Figure 7:**
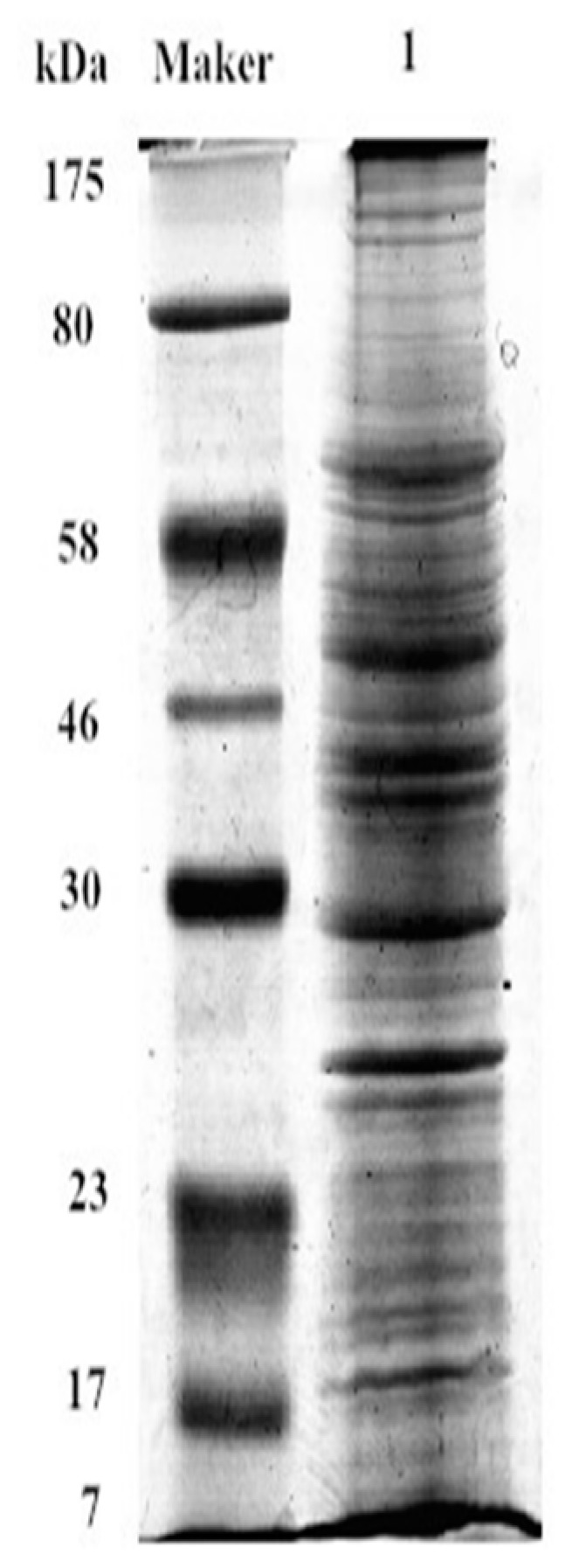
SDS-PAGE of IPEC-J2 membrane proteins. Maker, is a standard that is used to identify the approximate size of a molecule run on a gel during electrophoresis; Lane 1 represents membrane proteins, SDS-PAGE.

The extracted cell membrane proteins in IPEC-J2 were shown in Figure 7. Database searches with the peptide masses resulted in positive identification for 67 differential SBA associated proteins (Table 1). Integrins did not appear in the results of SBA-binding proteins. However, one integrin associated proteins: α-actinin-2 (ACTN2) was detected in the SBA-binding proteins. These results suggested that even there was no direct physical connection between integrins and SBA, ACTN2 may be one linking protein to connect SBA with integrins.

### 2.7. The Relationship between SBA, Integrins and ACTN2

**Figure 8:**
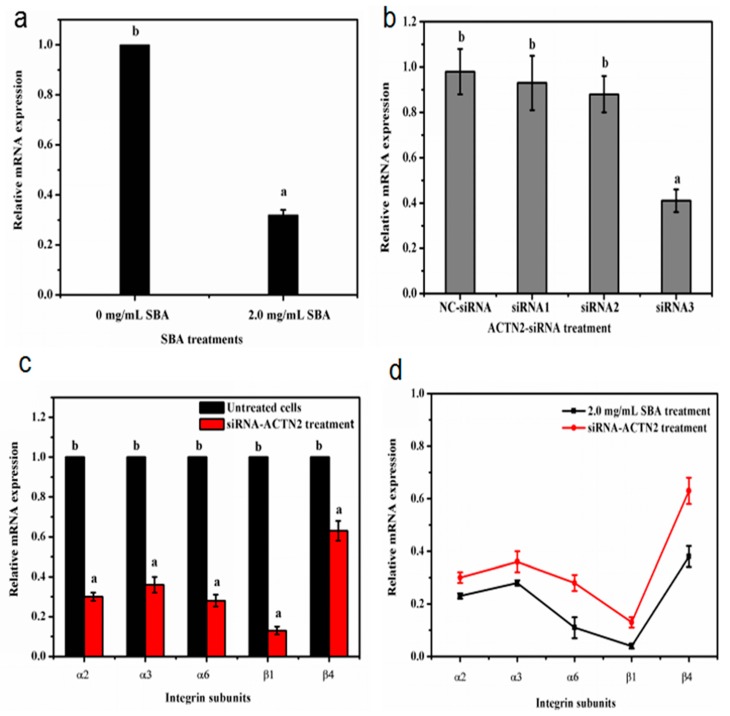
(**a**–**d**) The relationship between SBA, integrins and ACTN2. (**a**) Effects of SBA on mRNA expression of *ACTN2* in IPEC-J2. The cells were treated with 0 or 2.0 mg/mL SBA for 24 h, mRNA levels of *ACTN2* were analyzed by qRT-PCR; (**b**) Gene silencing efficiency of siRNA-*ACTN2* in IPEC-J2. After transfection with different pairs Negative control (NC)-siRNA, siRNA1, siRNA2, siRNA3) of siRNA-*ACTN2* sequences for 48 h, mRNA expression levels of *ACTN2* in different siRNA treatments were assessed using qRT-PCR; (**c**) The relative mRNA levels of integrin α2, α3, α6, β1 and β4 in siRNA-*ACTN2* treatment. The cells were treated with control or *ACTN2*-siRNA3 for 24 h and mRNA expression of integrins were detected using qRT-PCR; (**d**) The gene expression trends of different integrins (α2, α3, α6, β1 and β4) in SBA and siRNA-*ACTN2* treatments. Means with different superscripts are significantly different in compare with its control. All values are presented as means ± SEM (*n* = 3).

α-actinin does not only work as an important cytoskeletal protein to connect with integrins, but also act as a signal regular protein [16]. To determine whether SBA affects the gene expression level of integrin by *ACTN2*, the relationship between SBA and *ACTN2* gene expression was firstly detected. The qRT-PCR results indicated that after being stimulated with 2.0 mg/mL SBA for 24 h, mRNA expression of *ACTN2* was lower by 68% compared with control (*p* < 0.05, Figure 8a).

Next, corresponding siRNA sequence of *ACTN2* were selected to reveal the relationship between ACTN2 and integrin gene expression. We transfected the IPEC-J2 with three pairs of *ACTN2*-siRNA sequences or negative control (NC) siRNA for 24 h. The efficiency of *ACTN2* in vitro silencing was confirmed by qRT-PCR. The results demonstrated that the mRNA level of *ACTN2* in the siRNA3 group was significantly lower by 59% (*p* < 0.05), compared with the control group. Such level was even lower than the mRNA expression in both siRNA1 and siRNA2 groups. While the *ACTN2* mRNA level in NC group had no significant (*p* > 0.05) difference when compared with the control (Figure 8b). Therefore, the sequence of *ACTN2*-siRNA3 was used for the following experiment as this treatment induced the lowest mRNA expression of *ACTN2* than the other *ACTN2*-siRNA treatment.

Then, the cells were treated with control or *ACTN2*-siRNA3 for 24 h and mRNA expression of integrins were detected using qRT-PCR. The results revealed that the mRNA levels of integrin α2, α3, α6, β1 and β4 were significantly decreased in *ACTN2*-siRNA3 treatment (*p* < 0.05, Figure 8c). Besides, the low trends of integrin gene expression were similar in SBA treatment and *ACTN2*-siRNA3 treatment and shown in Figure 8d.

These results indicated that SBA can decrease the gene expression of *ACTN2*. The decreased gene expression of *ACTN2* by siRNA technique declined mRNA levels of integrins (α2, α3, α6, β1 and β4). Such trends were similar to the decreased gene expression of integrin induced by SBA. Therefore, SBA can reduce the mRNA expression of integrins by down regulating the gene expression level of *ACTN2*.

## 3. Discussion

Mainly, we aimed to investigate the effects of SBA on cell proliferation and cell apoptosis in IPEC-J2. The digestive epithelium is a highly organized tissue, and it requires only 3–5 days to be completely replaced. The health condition of the digestive epithelial cell proliferation and apoptosis is closely related to the nutrient digestion and absorption. In the current study, the cell proliferation of IPEC-J2 was lowered by SBA, indicating the damage effects of SBA on intestinal tract health. Bakke-McKellep et al. [17] found the same trend in Atlantic salmon, and this result was consistent with the research of Pan et al. [3] in IPEC-J2. In addition, we found that SBA can also induce cell apoptosis by increasing the fraction of caspase-3 positive apoptotic cells and lowering the mRNA expression of Bcl-2. Caspase-3 is a frequently activated protease in mammalian cell apoptosis [18]. Caspase-3 is involved in many apoptotic pathways, such as mitochondrial, death receptor pathways and endoplasmic reticulum stress pathway. Recently, Luft, et al. [19] and Nagayama and Tatsuno [20] judged apoptosis as by determination of the fraction of cells positive for active caspase-3. Bcl-2 is a proto-oncogene, which can inhibit apoptosis, mainly because Bcl-2 regulates a variety of cell proliferation- and apoptosis-related protein activity [21,22]. Kalashnikova et al. [23] indicated that nanoparticle delivery of curcumin induces cellular hypoxia and ROS-mediated apoptosis via modulation of Bcl-2 in human neuroblastoma. The dose of the SBA (2.0 mg/mL) was selected as we previously recommended [15], since this concentration has a significant inhibition rate on IPEC-J2 cell proliferation. Even there was no direct evidence to prove the effect of SBA on the apoptosis of intestinal epithelial cells, the results of Pusztai et al. [24] indirectly reflected the effect of SBA on the apoptosis of rat intestinal cells. Additionally, other plant lectins can also cause cell apoptosis. For example, Galectin-8 can bind to integrins, inhibit cell adhesion, and induce apoptosis [25]; Lectin of *Kaempferia rotunda* rhizome increases the percentage of the apoptosic cells in Ehrlich ascites carcinoma cells [26].

Integrins are crucial to cell survival and protect anchored cells against serum starvation-induced apoptosis. When integrin-mediated cell-matrix attachment is disrupted in epithelial and endothelial cells, cell apoptosis can be induced [27]. In the present research, we found that integrins played an important role in IPEC-J2 apoptosis, functional inhibition of any integrin inhibitors increased the percentage of the apoptotic cells. Pan et al. [15] have demonstrated that both integrin inhibitors and SBA lowered IPEC-J2 cell proliferation through the perturbation of cell cycle progression, and integrins were involved in the SBA-induced cell cycle progression alteration. Therefore, we hypothesized that integrin may be also involved in SBA induced IPEC-J2 cell apoptosis. To elucidate whether SBA affect cell apoptosis through the integrins in IPEC-J2, SBA was added to the integrin inhibitor treated cells to observe whether SBA still induced cell apoptosis in addition of integrin inhibitors. The results showed that the SBA could not induce cell apoptosis in addition of integrin inhibitors, indicating that integrins α2, α3, α6, β1 and β4 were involved in SCA in IPEC-J2. This may be closely related to the biological function of integrins in cell apoptosis. For example, integrin β4 is vital to the cell survival and prevents the cell apoptosis in primary cultured mouse neurons [28]; integrin α6 is an important protein to protect the primordial germ cells from apoptosis in the mouse [29].

In our research, the relationship between SBA and mRNA expression of integrins in IPEC-J2 was further identified in this research. After being stimulated by SBA, the mRNA expression levels in integrin subunits (α2, α3, α6, β1 and β4) were significantly lower than the control. Integrin subunits play a vital role in many types of biological cell proliferation and apoptosis [12], and the lowered gene levels of integrins can influence cell proliferation and cell apoptosis. For example, mammary epithelial proliferation is controlled through a signaling mechanism that demanded β1-integrin subunit, since the deletion of the β1-integrin gene inhibits cell proliferation in mammary epithelial cells [30]. Down-regulation of integrin α2 gene leads to decrease the capacity of osteoblast proliferation [31]. The reduction of α6 in mouse breast cancer cells can also inhibit cell proliferation [32]. Chung and Mercurio [33] reported that the deletion of α6β1 integrin in breast carcinoma cells induces cell apoptosis and decreases the transfer ability of the cells. β4 integrin is required for hemidesmosome formation, cell adhesion and cell survival, knockdown of β4 induces apoptosis in rat epithelial cells and Schwann cells [34]. Therefore, these results indicated that, SBA may inhibit IPEC-J2 proliferation and induce cell apoptosis as SBA lowered gene expression of integrin subunits.

Since SBA can significantly affect the gene expression in relation to integrins in IPEC-J2, another aim of this research was to determine the connection type between SBA and integrins, in other words, to prove by which pattern can SBA affect mRNA expression of integrins. The results showed that integrins had no direct association with SBA, which was different from Hadari et al. [25], who indicated that Galectin-8, termed S-type lectins, can bind to integrin α3, α6 and β1 and finally inhibit cell adhesion and induce cell apoptosis. This conflicting data from Hadari’s to our research may be ascribed to the different actions of different lectins’ kinds to interact with their ligands [35]. In addition, lectins have their striking biological activities depending on lectin species, animal species, developmental stage and other factors [36]. Therefore, additional research is needed to study the connection type between different types of lectins and integrin family members.

For further identification whether SBA influenced integrins gene expression by an indirect way, integrin-associated proteins in SBA-binding proteins in the IPEC-J2 cell membrane were analyzed. In the present study, one integrin-associated protein was identified in SBA-binding proteins, family members of α-actinin, ACTN2. α-actinin is a major component in isolated plasma membranes in HeLa cells, mouse myeloma cells, and gerbil fibroma cells [37]. The direct association between α-actinin and integrins has been reasonably well established. For example, the central rod domain of α-actinin consists of four spectrin-like repeats, which binds to the cytoplasmic tail of integrin β1 subunits [38]. A high affinity binding sites on the integrin β1 cytoplasmic domain (residues F743-A753) are binding to the central repeats of α-actinin [39]. In this research, SBA lowered *ACTN2* and integrin mRNA levels. The gene expression level of these five integrin subunits was decreased after *ACTN2* gene silencing by RNA interference technique. Besides, the reduced trends of integrin gene expression were similar in SBA treatment and *ACTN2*-siRNA3 treatment. The related research demonstrated that α-actinin is not only one integrin-associated protein, but also a signal regular protein [16]. For example, α-actinin plays a potential role in regulating integrin αIIbβ3 activation [40]. Furthermore, α-actinin is also the main link transmitting force between integrins and the cytoskeleton in mature adhesion [41]. Alteration of α-actinin expression levels can also affect integrin functions and signal transduction [42]. Therefore, ACTN2 may be not only one of the proteins that binds to SBA and integrin, but also can transfer the signal of SBA to integrin. In other words, SBA may reduce the mRNA expression of integrins by down regulating the gene expression level of *ACTN2*.

The gastrointestinal epithelial cells in pigs are the most similar models to the human gastrointestinal epithelial cells [43]. Thus, using of SBA in high levels for human nutrition could exhibit relevant level of gastrointestinal cell apoptosis. The residual SBA (undigested amount, %) in different animals and different intestinal digesta was analyzed according to the following model: *y* = A*i* + D*j* + A*i*D*j* + e*ij* (where *y* is the measurement of residual SBA index; A*i* is the experimental results produced by different species of animals; D*j* is the experimental results produced by different intestinal segments; A*i*D*j* is the interaction between different species of animals, and different intestinal segments; e*ij* is the random error) [44]. The concentration of SBA in raw soybean is ranged between 10 to 20 mg/g, depending on the soybean species [45], and the residual SBA in pig jejunum was 17% [44]. Thus, the high levels of soybean (like natural grazing) or soybean products contained in diets could lead to apoptotic effect on the intestinal epithelial cells, as a result of the action of SBA.

## 4. Materials and Methods

### 4.1. Cell Culture

IPEC-J2 (Porcine intestinal columnar epithelial cell line) was cultivated by applying standard cell culture techniques at 37 °C with 5% CO_2_ in Dulbecco’s Modified Eagle Media: Nutrient Mixture F-12 (DMEM/F12) medium (Gibco, Waltham, MA, USA), supplemented with 10% (*v*/*v*) fetal bovine serum (FBS, Gibco) and 1% (*v*/*v*) penicillin-streptomycin (Sigma, St. Louis, MO, USA). The cell culture medium was exchanged every 2 days.

### 4.2. Cell Morphological Observation

IPEC-J2 was seeded at 5 × 10^4^ cells/cm^2^ in 6-well plates and cultured for 80% confluence. Then the cells were cultured with 0, 0.125, 0.25, 0.5, 1.0 or 2.0 mg/mL SBA for 24 h. Cell morphology in different treatments was observed by contrast microscopy (Olympus, Tokyo, Japan).

### 4.3. Cell Proliferation Assays

IPEC-J2 was seeded into 96-well plates at a density of 5 × 10^3^ cells per well for 80% confluence. Then the cells were cultured for 24 h in the presence of SBA (Sigma), dissolved in DMEM/F12 medium at concentration of 0, 0.125, 0.25, 0.5, 1.0 or 2.0 mg/mL. The media were discarded and the cell proliferation was quantified by MTT assay according to the manufacturer’s instructions (Sigma). The plates were read using a multiplate reader (Multiskan FC, Thermo Scientific, Waltham, MA, USA) at 570 nm wavelength.

### 4.4. Cell Apoptosis Assessment by Flow Cytometry

Upon reaching 80% confluence, the cells were treated with 0, 0.125, 0.25, 0.5, 1.0, or 2.0 mg/mL SBA for 24 h. FITC active caspase-3 apoptosis kit (BD Pharmingen, La Jolla, CA, USA) was used to determine the cell apoptosis by FCM according to the manufacturer’s instructions. In brief, the cells in different treatments were washed with cold phosphate buffer solution (PBS), then resuspend cells in BD Cytofix/Cytoperm™ solution at a concentration of 1 × 10^6^ cells/0.5 mL. After incubation for 20 min on ice, BD Cytofix/Cytoperm™ solution was discarded and the cells were washed twice with BD Perm/Wash™ buffer at room temperature. The cells were then incubated with BD Perm/Wash™ buffer plus antibody at room temperature for 30 min. Each test was washed in 1.0 mL BD Perm/Wash™ buffer, then the test was suspended in 0.5 mL BD Perm/Wash™ buffer. A minimum of 1 × 10^4^ cells were collected and analyzed using FCM. The optimal concentration of SBA was selected for the next experiment.

### 4.5. qRT-PCR

IPEC-J2 was grown on glass tissue culture flasks with the density of 1 × 10^5^ cells per flask (25 cm^2^, NUNC, A/S, Roskilde, Denmark) for 80% confluence. After treatment with 0 (control) or optimal concentration of SBA for 24 h, total RNA was extracted using Trizol reagent (Takara, Shiga, Japan) according to the manufacturer’s protocol. A Nanodrop 2000 spectrophotometer (Thermo Scientific) was used to determine the yield and purity of the total RNA. 1 μg of total RNA was used for cDNA synthesis which was carried out in 20 μL reaction system using cDNA synthesis kit (Takara) following manufacturer’s instruction.

qRT-PCR was performed using the SYBR Premix Ex Tap II (Tli RNaseH Plus, Takara). Thermal cycling consisted of initial denaturation for 30 s at 95 °C RT stage, followed by 5 s at 95 °C, then 30 s at 60 °C for 40 cycles, then 15 s at 95 °C, 30 s at 60°C and 15 s at 95 °C. Cycle threshold (*C*_t_) values were measured and calculated by the Sequence detector software. Relative amounts of mRNA were normalized to GAPDH and calculated with the software program Microsoft Excel. The relative mRNA expression levels of different genes were calculated using the formula: 2^−ΔΔ*C*t^ [46], in which ΔΔ*C*_t_ = ΔE − ΔC, and ΔE = *C*_t sample_ − *C*_t GAPDH_ and ΔC = *C*_t control_ − *C*_t GAPDH_. The primer sequences with a concentration of 10 µM for different genes and GAPDH are shown in Table 2.

### 4.6. Integrin Functional Inhibition Test

According to our previous study [15], 10 µg/mL of different integrin functional inhibitors (α2: MAB1950Z; α3: MAB1952P; α6: MAB1378; β1: MAB1959; or β4: MAB2058, Millipore, MA, USA) and SBA with optimal concentration were selected in integrin inhibitor test. Brifely, the cell proliferation rate in the first effective concentration of α2 treatment was 10 µg/mL; in α3, 10 µg/mL; in α6, 5 µg/mL; in β1, 5 µg/mL and in β4 was 10 µg/mL. To ensure the consistency of experimental conditions, 10 µg/mL was selected as the final optimal concentration for each subunit in the subsequent integrin inhibitor experiments. The IPEC-J2 was seeded into 6 well plates for 80% confluence and then divided into twelve groups as shown in Table 3. The cell apoptosis in different groups were measured using FCM and conducted as described before (in Section 4.4).

### 4.7. Effects of SBA on Integrin Gene Expression by qRT-PCR

IPEC-J2 was grown on glass tissue culture flasks with the density of 1 × 10^5^ cells per flask (25 cm^2^, NUNC, Denmark) for 80% confluence. The relative mRNA expression levels of integrins were calculated using the formula: 2^−ΔΔ*C*t^ [46]. The primer sequences with a concentration of 10 µM for integrins α2, α3, α6, β1, β4 and GAPDH are shown in Table 2.

### 4.8. Preparation of Cell Membrane Protein Samples

IPEC-J2 was grown on glass tissue culture flasks for complete differentiation. Then cell membrane proteins of IPEC-J2 were prepared using a Native Membrane Protein Extraction Kit (Calbiochem, Darmstadt, Germany) according to manufacturer’s instructions. The concentration was determined using BCA protein assay kit (Thermo Scientific) and protein samples were stored at −80 °C for the later use.

### 4.9. SDS-PAGE

The extracted IPEC-J2 membrane proteins were isolated by SDS-PAGE. Cell membrane proteins were re-suspended in loading buffer (CWBIO, Beijing, China) with the ratio of 4:1 (sample weight to buffer weight). After centrifugation, the supernatant was heated to 95 °C for 3 min. Then the boiled samples were kept in iced condition before loading into the gel. Both 5% stacking gel and 10% separation gel were prepared for separating the proteins. 10 μL of membrane proteins, as well as protein marker (BioLab, Beijing, China) were applied on the gel. The SDS-PAGE was performed at 80 V through the stacking gel and 150 V through the separation gel. After fixation, these proteins were stained with Coomassie brilliant blue at room temperature.

### 4.10. Co-Immunoprecipitation

Co-Immunoprecipitation experiment was conducted to separate all the specific proteins that specially combined with SBA on IPEC-J2 cell membrane. Co-Immunoprecipitations were carried out using Dynabeads Co-Immunoprecipitation Kit (Invitrogen, Carlsbad, CA, USA). The conditions of all buffers provided in this kit were conducted strictly in accordance with the manufacturer’s instructions. Prior to immunoprecipitation, 50 µg of SBA (Sigma) was mixed with beads, and incubated on a roller at 37 °C overnight to make sure the fluid in the tube mix well.

After washing with wash buffer, 1.5 mg prepared SBA-coupled beads were transferred to a fresh tube. Then the SBA-coupled beads were re-suspended in 1.0 g cell membrane samples and incubated on a roller at 4 °C for 30 min. Then the beads were finally re-suspended in EB solution and incubated on a roller at room temperature for 5 min. The immunoprecipitate was centrifuged and the supernatant which contained SBA-binding protein complexes was transferred to a clean tube.

### 4.11. Liquid Chromatography Coupled to Tandem Mass Spectrometry (LC-MS-MS (Q-E)) Detection

After separation by co-immunoprecipitation, all SBA associated proteins were detected by Beijing Protein Innovation CO., Ltd. (Beijing, China) and analyzed using an electro-spray ionization tandem mass spectrometry LC-MS-MS (Q-E). MASCOT protein scores (based on combined MS and MS/MS spectra) > 22 were considered statistically significant (*p* < 0.05). The individual MS/MS spectrum, with a statistical significant (confidence interval > 95%) iron score was accepted.

### 4.12. In Vitro Gene Silencing of ACTN2

For further investigation of the relationship between α-actinin-2 (ACTN2) and integrin subunits, siRNA technique was employed in this study. Cells were seeded in a 6-well plate (5 × 10^4^ per well), after upon reaching 80% confluence, they were used for gene silencing experiment. According to the gene sequences of *ACTN2* specific siRNA in Genbank, we designed three pairs of *ACTN2*-siRNA (Changsha Yingrun Biotechnology, Changsha, China) and their sequences are shown in Table 4.

*ACTN2* silencing experiment was divided into five groups: control (untreated cells), negative control (NC), siRNA1 group, siRNA2 group and siRNA3 group. After being diluted in Opti-MEM Medium (Invitrogen), siRNAs and Lipofectamine were mixed and conducted following the manufactural protocol of LipofecAMINE RNAiMAX Reagent (Invitrogen) and cultured at 37 °C for 24 h.

In vitro gene silencing efficacies of the *ACTN2*-siRNA complexes were evaluated by qRT-PCR. The target sequences for *ACTN2* is shown in Table 2. Silencing efficacies of different pairs of *ACTN2*-siRNA sequences were compared, and then screened one siRNA sequence with the best silencing efficiency for the next experimental process.

### 4.13. Determination of the Interaction between SBA, ACTN2 and Integrins from the Gene Levels by qRT-PCR

Upon reaching 80% confluence, the cells were stimulated with 0 or optimal concentration of SBA for 24 h, total RNA in different SBA treatments were prepared and qRT-PCR was conducted to determine the roles of SBA on mRNA expression of *ACTN2*.

IPEC-J2 was seeded in a 6-well plate (5 × 10^4^ per well) for 80% confluence. Then the cells were treated with 0 or optimal sequences of *ACTN2*-siRNA for 24 h. Their total RNA was extracted and qRT-PCR was used to detect the mRNA levels of integrins in different treatments to explore the relationship between *ACTN2* and gene expression of integrins.

### 4.14. Statistical Analysis

Each experiment was repeated at least for three times and numerical data were presented as mean ± SEM. Student’s *t*-test was used to compare the data between two groups. Data among three or more groups were analyzed with ANOVA followed by the least significant difference (LSD) tests, SPSS Statistics Base 17.0 [47], *p <* 0.05 was considered significant.

## 5. Conclusions

Integrins were important for the process of IPEC-J2 apoptosis and SCA. Although there was no direct connection between SBA and integrins, ACTN2 acted as a bridge to connect SBA and integrins. SBA may reduce the mRNA expression of integrins by down regulating the gene expression level of *ACTN2*. These results provided an important evidence and theoretical basic for revealing the anti-nutritional mechanism of SBA, and provided a new way to find the nutritional regulation measures to alleviate the SBA anti-nutritional mechanism. However, whether ACTN2 is the only protein for connecting SBA to integrins is not clear, and still needs to further exploration.

## Figures and Tables

**Table 1:** Identification of SBA receptors by Q–E analysis.

No	Name	Accession No.	Score	m_w_/PI	Sequence Coverage
1	KRT1	F1SGG3	1973	65.381/8.18	14%
2	KRT14	F1S0L1	1309	51.811/4.94	40%
3	KRT10	I3LDS3	1265	58.243/4.93	17%
4	KRT5	F1SGG6	870	63.668/7.74	20%
5	KRT75	F1SGI7	697	65.354/9.27	12%
6	KRT84	F1SGI2	619	54.121/6.12	5%
7	LOC100737483	F1SGG9	564	60.375/8.53	19%
8	MYH13	71SS66	550	223.705/5.54	1%
9	KRT2A	A5A759	539	66.510/7.47	8%
10	KRT3	I3LDM6	525	65.497/8.03	11%
11	PYGM	F1RQQ8	507	97.665/6.65	7%
12	LOC100302368	F1SRS2	464	26.563/6.85	26%
13	KRT8	F1SGG2	440	54.394/5.70	7%
14	CKM	I3LBJ8	411	43.114/6.91	10%
15	ALB	F1RUN2	409	71.621/5.98	8%
16	Jup	Q8WNW3	399	82.539/5.75	24%
17	ACTA1	P68137	368	42.366/5.23	17%
18	KRT15	F1S0K2	317	49.501/4.87	8%
19	l-lactate dehydrogenase	D2SW96	275	36.582/8.18	6%
20	Myosin-4	Q9TV62	245	224.0.1/5.60	2%
21	LOC100519531	F1RU49	174	103.873/5.31	5%
22	SLC25A4	F1RZQ6	173	33.233/9.82	14%
23	GFAP	F1RR02	166	49.463/5.32	4%
24	HUMMLC2B	A1XQT5	143	19.066/4.82	10%
25	CS	Q0QEK1	142	24.886/6.59	5%
26	ACTC1	B6VNT8	141	42.334/5.23	18%
27	MYH4	Q9TV62	139	224.010/5.60	1%
28	DSP	F1RW75	129	310.122/6.49	4%
29	LOC407246	I3LL80	108	36.884/5.57	11%
30	LOC100515788	F1RII5	107	15.682/7.93	6%
31	ATP2A2	P11607	93	116.371/5.23	3%
32	PCBP1	I3LEC2	93	37.991/6.66	5%
33	LDHB	F1SR05	89	37.108/5.45	5%
34	GAPDH	G3CKJ2	87	29.621/9.29	8%
35	MB	P02189	85	17.074/6.75	10%
36	Uncharacterized protein	F1SIX1	84	17.814/5.82	15%
37	PKM	F1SHL9	82	59.256/7.95	2%
38	HUMMLC2B	Q5EEL9	81	10.704/4.80	16%
39	AK1	P00571	75	21.739/8.38	6%
40	BLVRB	I3LQH7	75	22.400/6.35	10%
41	TMEM38A	F1S9V9	74	29.180/8.79	5%
42	tnnt3	Q75NG6	70	29.811/7.74	6%
43	MYH7	F1S9D6	67	223.795/5.59	0%
44	HUMMLC2B	Q5EEL9	59	10.704/4.80	16%
45	YWHAG	F2Z4Z1	55	28.456/4.80	13%
46	TPI1	D0G7F6	51	26.879/6.54	5%
47	SOD2	F1SB60	49	24.923/8.59	9%
48	Uncharacterized protein	F1RWF5	49	28.518/5.50	7%
49	MLC2V	A1XQV9	45	16.862/4.57	
50	ENO2	I3LCN1	44	47.438/4.93	5%
51	LOC100621642	I3LJ52	39	28.128/6.69	3%
52	ANXA2	P19620	38	38.795/6.49	13%
53	KRT18	A8D349	37	18.038/4.63	10%
54	PCBP2	F1SFQ4	37	37.606/6.60	5%
55	Beta-actin	A0SNU5	37	29.678/5.50	11%
56	PKP1	I3LGN8	36	53.569/9.12	6%
57	ACTN2	F1RHL9	34	104.237/5.31	2%
58	Glyceraldehyde-3-phosphate dehydrogenase	A0SNU7	34	28.035/6.77	5%
59	HADH	P00348	32	34.197/9/02	11%
60	PFMK	Q1HL06	30	82.443/8.57	2%
61	ANK2	F1S146	28	428.715/4.93	0%
62	CA3	Q5S1S4	28	29.679/7.72	9%
63	ATP2A1	F1RFH9	27	110.403/5.17	3%
64	METTL3	F1S8J8	26	65.501/6.12	1%
65	CABLES1	F1SBB7	26	64.246/9.61	1%
66	CKMT2	Q2HYU1	25	47.961/8.47	3%
67	VDAC1	Q9MZ16	24	30.882/8.62	7%

Accession number in the NCBInr database. m_w_ means molecular weight; PI means isoelectric points. Peptide score is shown as −10log (*p*), where P is the probability that the observed match is a random event. Individual ions scores >22 indicate identity or extensive homology (*p* < 0.05). Sequence coverage is shown with the respect to match tryptic digest fragments.

**Table 2:** Primer sequences of different genes.

Gene	Accession No.	Forward Primer	Reverse Primer
Bcl-2	NM_214285.1	TGTGCGTGGAGAGCGTAGA	TAGGTGGTCATTCAGGTAAGTGG
α2 (ITGA2)	NM_001244272	CGCAGATTACGCTCCTCAAAA	GCTGAACAACAGTCCCACTCC
α3 (ITGA3)	XM_005668879	AGCGTCCCCACCATCAA	CTCACAGCCACAAGCACCA
α6 (ITGA6)	XM_005657544	TGTGACTGTGTTTCCCTCAAAGA	AGCATCAAAATCCCAGCAAGA
β1 (ITGB1)	XM_003131195	TGAGTGCAACCCCAACTACAC	CAGACCCCACATTCACAGACA
β4 (ITGB4)	NM_213968.1	CAGGGCTACAGCGTGGAGTA	TAGGAGTGGTTGGGCAGAAGA
*ACTN2*	NM_001243666	GGCATACGGCAAAGAGCA	GGCGTCGTGATAGTCCAGTT
GAPDH	NM_001206359	TGCACCACCAACTGCTTGGC	GGCATGGACCGTGGTCATGAG

**Table 3:** Divided groups in integrin inhibitor experiment.

Treatment Number	SBA Level	Integrin Inhibitor Type
1 (control)	-	-
2	SBA	-
3	-	α2 inhibitor
4	SBA	α2 inhibitor
5	-	α3 inhibitor
6	SBA	α3 inhibitor
7	-	α6 inhibitor
8	SBA	α6 inhibitor
9	-	β1 inhibitor
10	SBA	β1 inhibitor
11	-	β4 inhibitor
12	SBA	β4 inhibitor

**Table 4:** Target siRNA sequences of *ACTN2*.

Gene Name	Forward Primer	Reverse Primer
*ACTN2*-siRNA1	GCACUCAGAUCGAGAACAUTT	AUGUUCUCGAUCUGAGUGCTT
*ACTN2*-siRNA2	GCAUGAUCUGGACCAUUAUTT	AUAAUGGUCCAGAUCAUGCTT
*ACTN2*-siRNA3	GGUUCUUGCUGUGAAUCAATT	UUGAUUCACAGCAAGAACCTT
NC-siRNA	GACTTCATAAGGCGCATGC	GCATGCGCCTTATGAAGTC

## Supplementary Materials

Supplementary materials can be found at http://www.mdpi.com/1422-0067/19/2/587/s1.

## Abbreviations

ACTN2	α-Actinin-2
FCM	Flow cytometry
IPEC-J2	Porcine intestinal columnar epithelial cells
LC-MS-MS	Liquid chromatography coupled to tandem mass spectrometry
MTT	3-(4,5-Dimethylthiazol-2-yl)-2,5-diphenyltetrazolium bromide
NCA	Normal cell apoptosis
qRT-PCR	Quantitative real-time polymerase chain reaction
SBA	Soybean agglutinin
SCA	SBA-induced cell apoptosis
