# Supplementary material for: Integrins Were Involved in Soybean Agglutinin Induced Cell Apoptosis in IPEC-J2
Source: Int J Mol Sci. 2018 Feb 16;19(2):587. doi: 10.3390/ijms19020587 (PMC5855809; doi:10.3390/ijms19020587)
Supplement: Supplementary file 1 [file ijms-19-00587-s001.pdf]

International Journal of
Molecular Sciences

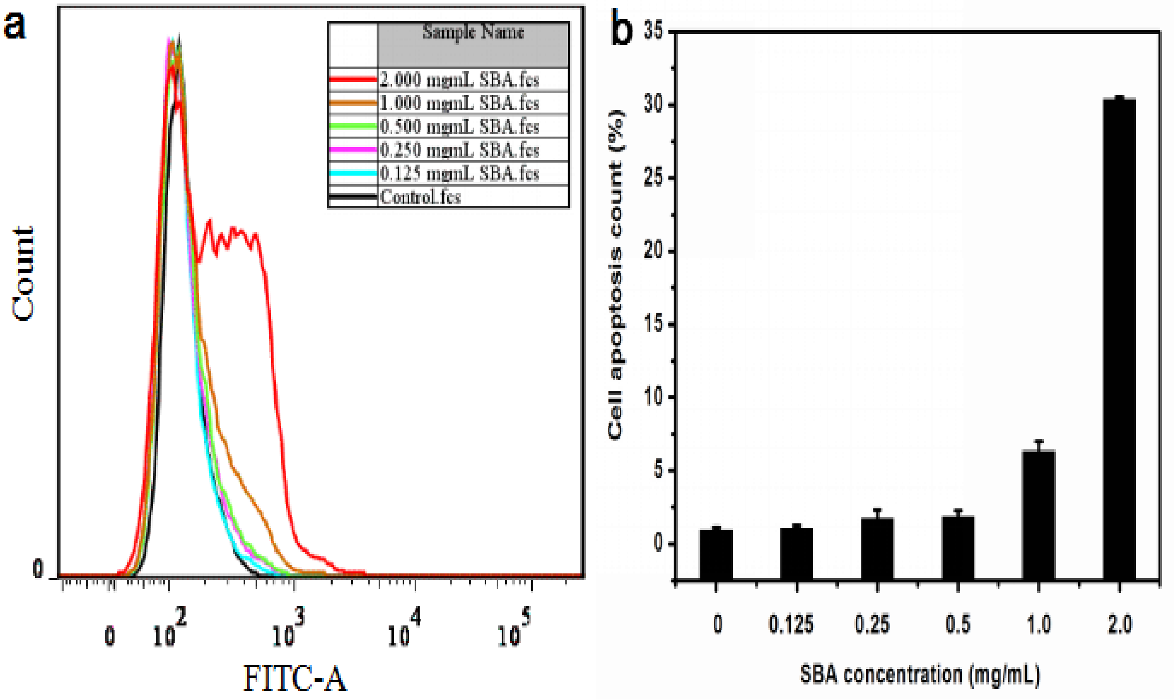

Figure S1. Proportions of IPEC-J2 cell apoptosis in different SBA treatments. IPEC-J2s were treated with different concentrations (0, 0.125, 0.25, 0.5, 1.0, or 2.0 mg/mL) of SBA for 24 h. (**a**). Curve fitting of apoptotic data in different SBA treatment; (**b**) Analysis of the percentage of the apoptotic cells in different treatments. Data are represented as mean ± SEM (*n* = 3).

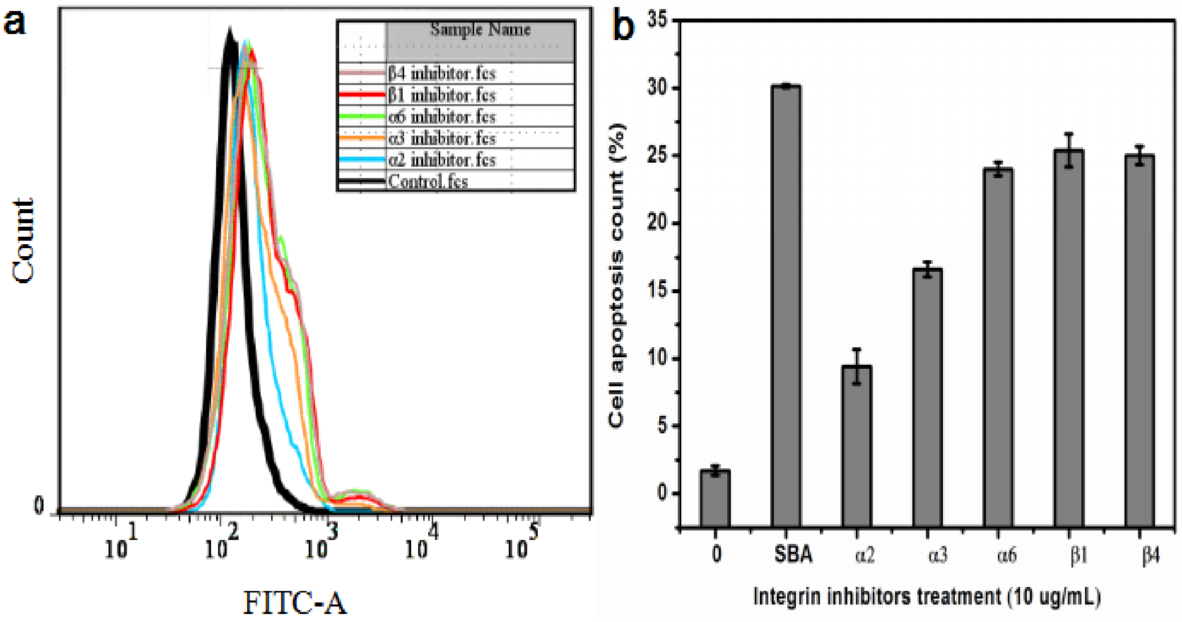

Figure S2. Functions of integrin inhibitors on IPEC-J2 cell apoptosis. IPEC-J2s were treated with 10 μg/mL of different integrin (α2, α3, α6, β1 or β4) inhibitors for 24 h. (**a**). Curve fitting of apoptotic data in different integrin inhibitor treatment; (**b**) Analysis of the percentage of the apoptotic cells in different treatments. Data are represented as mean ± SEM (*n* = 3).

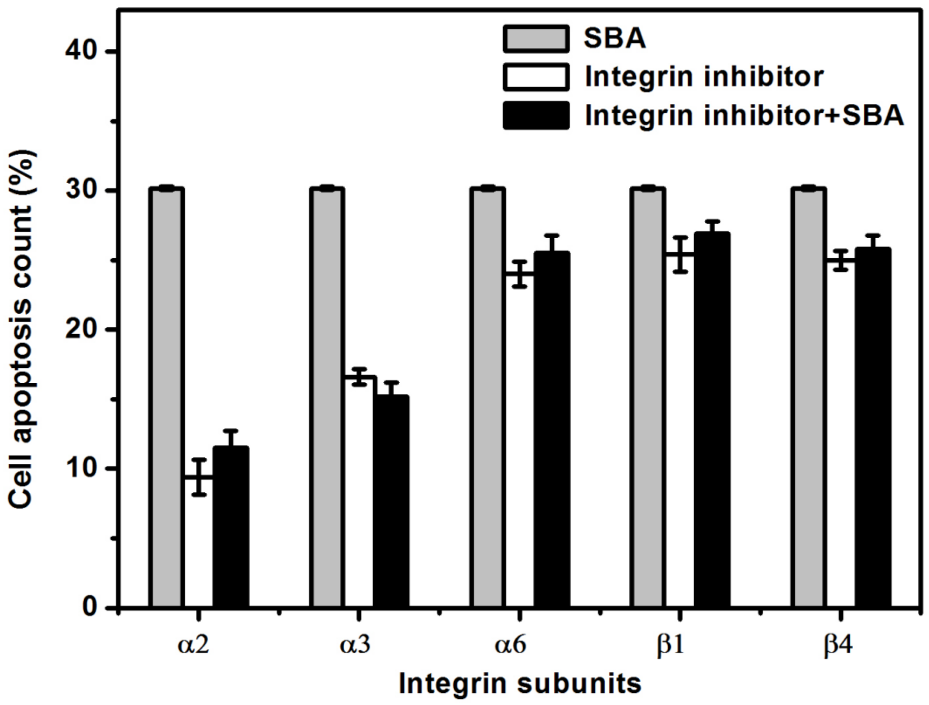

Figure S3. The roles of integrin inhibitors in SBA-induced cell apoptosis. IPEC-J2s were treated with 10 μg/mL of integrin inhibitors (*α*3, *α*6, β1 or β4) or 10 μg/mL integrin inhibitor (*α*3, *α*6, β1 or β4) + 2.0 mg/mL SBA for 24 h. The apoptotic data are presented as the mean ± SEM (*n* = 3).
